# The impact of the private sector co-payment mechanism (PSCM) on the private market for ACT in Nigeria: results of the 2018 cross-sectional outlet and household market surveys

**DOI:** 10.1186/s12936-021-04039-9

**Published:** 2022-02-12

**Authors:** Hannah M. Edwards, Rubaiyath Sarwar, Parvez Mahmud, Shekarau Emmanuel, Kolawole Maxwell, James K. Tibenderana

**Affiliations:** 1 Malaria Consortium Headquarters, 244-254 Cambridge Heath Rd, London, E2 9DA UK; 2 Innovision Consulting Private Limited, Level 3 & 4 House 26 Road 6 Baridhara J Block Pragati Sarani, Dhaka, 1212 Bangladesh; 3 Case Management Branch, National Malaria Elimination Programme, First Floor, Abia House, Central Business District, Abuja, Nigeria; 4 Malaria Consortium Nigeria, 33 Pope John Paul Street, Off Gana Street, Maitama, Abuja-FCT, Nigeria

**Keywords:** Private sector healthcare, Case management, Health economics, Malaria control, Informal health workers, Private sector engagement, Targeted subsidy, Artemisinin-based combination therapy, Private sector co-payment mechanism

## Abstract

**Background:**

The private sector plays a large role in malaria treatment provision in Nigeria. To improve access to, and affordability of, quality-assured artemisinin-based combination therapy (QA-ACT) within this sector, the Affordable Medicines Facility-Malaria began operations in 2010 and transitioned to a private sector co-payment mechanism (PSCM) until 2017. To assess the impact of the scheme on the ACT market, cross-sectional household and outlet surveys were conducted in 2018 to coincide with the final stockages of ACT medicines procured under the PSCM.

**Methods:**

An outlet survey was conducted targeting private pharmacies and Proprietary and Patent Medicine Vendors (PPMVs) across different regions of Nigeria to assess supply-side market factors related to availability and cost of anti-malarials, including artemisinin-based combinations subsidised under the PSCM (called green leaf ACT on account of their green leaf logo) and those not subsidised (non-green leaf ACT). A concurrent household survey was conducted to determine demand-side factors related to treatment-seeking practices, ACT brand preference and purchase decision. Data were compared with previous ACTWatch surveys to consider change over time.

**Results:**

Availability of artemisinin-based combinations increased significantly over the PSCM period and was almost universal by the time of the 2018 market survey. This increase was seen particularly among PPMVs. While the cost of green leaf ACT remained relatively stable over time, the cost of non-green leaf ACT reduced significantly so that by 2018 they had equivalent affordability. Unsubsidised brands were also available in different formulations and dosages, with double-strength artemisinin-based combination reported as the most frequently purchased dosage type, and child artemisinin-based combinations popular in suspension and dispersible forms (forms not subsidised by the PSCM).

**Conclusions:**

The PSCM had a clear impact on increasing not only the reach of subsidized QA brands, but also of non-subsidised brands. Increased market competition led to innovation from unsubsidised brands and large reductions in costs to make them competitive with subsidised brands. Concerns are drawn from the large market share that non-QA brands have managed to gain as well as the continued market share of oral artemisinin monotherapies. Continued monitoring of the market is recommended, along with improved local capacity for QA-certification and monitoring.

**Supplementary Information:**

The online version contains supplementary material available at 10.1186/s12936-021-04039-9.

## Background

Appropriate and timely case management is an essential component in any successful malaria control programme. Over recent years there has been emphasis on promotion of malaria diagnostic testing to replace presumptive treatment, followed by appropriate treatment with artemisinin-based combination therapy (ACT) in response to a positive test result [1]. Point-of-care rapid diagnostic tests (RDTs) have made this increasingly possible since they are cheap and easy to administer by healthcare workers, community volunteers or private providers with minimal training, while quality-assured ACT (QA-ACT) ensure thorough elimination of the parasite from the bloodstream [2]. Poor quality or incorrect drug regimens, including use of monotherapies, can lead to failure of treatment for the individual and increase drug resistance pressure on the parasite [3, 4].

Ensuring high coverage and implementation of this case management pathway has proved difficult in many endemic countries, however. Many populations most afflicted with malaria are in remote rural areas with limited access to health facilities [5–7]. These populations are often served by a variety of healthcare providers including public health facilities, private hospitals, pharmacies, drug stores and roaming traders [8]. While public health care provision can be controlled and monitored by national malaria control programmes, private channels are often unregulated and case management practices unsupervised. There is increasing recognition that in order to reach many population groups, engagement with the private sector is needed in order to harmonize diagnostic and treatment methods, reduce the prevalence of substandard and counterfeit drugs, and ensure the removal of mono-artemisinin drugs and other anti-malarials no longer recommended as first-line treatment [9, 10].

Nigeria, a country with a national malaria prevalence in children aged 6–59 months of 23% in 2018 (although with high heterogeneity between states), has a large private sector pharmaceutical market [11]. Nigeria was one of eight countries to be targeted with a financing mechanism called the Affordable Medicines Facility—Malaria (AMFm) funded by the Global Fund to Fight AIDS, Tuberculosis & Malaria (GFATM), launched in 2010 [12]. The aim of AMFm was to subsidise the cost of QA-ACT that had been through the World Health Organization (WHO) pre-qualification certification programme thereby increasing the use of pre-qualified ACT medicines (PQ-ACT) and crowding out monotherapies [13]. Specific interventions included i) negotiating price reductions from manufacturers to private-sector importers/first-line buyers (FLBs), ii) subsidising the cost of the ACT medicines from manufacturers to further reduce costs to FLBs, and iii) supporting interventions to promote appropriate ACT use, such as training and behaviour change communication for private sector vendors [14].

Ultimately, these interventions aimed to increase affordability of QA-ACT and, in turn, increase consumer access and uptake through the private sector [12]. The subsidised PQ-ACT medicines were marked with a ‘green leaf’ logo to aid identification and market promotion (herein referred to as “green leaf ACT”) [14]. An evaluation of the scheme in 2012 showed positive impact across most target countries in terms of increasing QA-ACT availability and market share, and in reducing QA-ACT price to consumers [14, 15]. Following this initial success, six of these countries, including Nigeria, transitioned the AMFm into a similar subsidy scheme called the Private Sector Co-payment Mechanism (PSCM) with operations in Nigeria running until 2017.

During the AMFm-PSCM implementation period, regular ACTWatch surveys were conducted in Nigeria that showed the availability of green leaf ACT increased from 8.7% in 2011 to 17.7% in 2015 across all private sector providers, though this was most marked among drug stores, also known as Proprietary and Patent Medicine Vendors (PPMVs), where availability rose from 49.4% to 78.8% [16]. As well as increases in availability, QA-ACT medicines were also found to have an increase in market share and a reduction in price to the consumers, findings that were echoed among other countries with the PSCM, including Kenya, Madagascar, Tanzania and Uganda [12]. The positive impact of ACT subsidies was further demonstrated by a 2015 meta-analysis of 12 quantitative studies across Africa and the Greater Mekong Subregion, which found that ACT subsidies increased ACT use among consumers; for every $1 decrease in price, ACT use increased by 24 percentage points among suspect malaria cases [17]. Since the last ACTWatch survey was carried out in Nigeria in 2015, there has not been a final assessment of the full PSCM period which terminated in 2018. Furthermore, the ACTWatch surveys only targeted health providers and outlets and thus only assessed supply-side market indicators; they did not provide information on market demand from consumers. To assess the state of the private-sector RDT and ACT market in Nigeria at the end of the PSCM a national outlet survey of private pharmacies and PPMVs was conducted along with a nationwide household survey to assess both supply and demand side impact of the PSCM. Whereas pharmacies are run by trained health professionals, a PPMV is defined as ‘a person without formal training in pharmacy who sells orthodox pharmaceutical products on a retail basis for profit’ [18]. These two outlet types play a significant role in diagnostic and treatment provision in Nigeria but are unregulated and thus are a key focus for private–public-partnership engagement. The surveys were also designed to provide a baseline assessment from which the market could be monitored once the subsidy scheme had been removed. Here, results of these surveys are presented specifically in relation to the impact on the private-sector ACT market, including impact on both the supply (outlet) and demand (household) sides of the market.

## Methods

Two cross-sectional surveys—an outlet survey and a household survey—were conducted nationwide in Nigeria in 2018 at the end of the PSCM. The aim was to assess both demand and supply side market systems via a quantitative approach.

### Supply side analysis: outlet survey

The objective of the outlet survey was to ascertain the current status of availability of ACT medicines, gaps in the market, market share and prices of different anti-malarial brands. The survey was targeted to both PPMVs and pharmacies in the private retail sector, and sampling was conducted to cover the six states of Nigeria representing the six geo-political zones (North-Central, North-East, North-West, South-South, South-West, South-East) as well as different malaria endemicities.

### Sampling strategy

Since the total number of providers was unknown, sample size was determined assuming the population size to be > 20,000 (or unknown), with a 50% response rate, 5% margin of error and 95% confidence level and a design effect of 2. This estimated a sample size of 768 outlets for the survey. All the states in Nigeria were segregated into four groups based on the prevalence of malaria to ensure representation across the four malaria endemic zones—Group 1: 0–10% prevalence, Group 2: 10–30% prevalence, Group 3: 30–50% prevalence, and Group 4: > 50% prevalence. The population size of each malaria endemic zone was calculated by summing the population size of all the states within each group. The sample size of outlets for each group was then split based on the ratio of the population in each group compared to the total population of all groups (Table 1).

**Table 1:** Outlet and Household survey sampling locations and sample size across selected groups, including planned sample size and actual sample size achieved

Outlet survey sample						
Malaria endemic group	# total states nationwide	2018 Population	% total population	Outlet sample size (planned)	Selected state(s)	Sample size achieved
Group 1: 0–10%	2	19,086,824	10	75	Imo	77
Group 2: 10–30%	24	114,617,062	59	449	Kogi Gombe Osun	406
Group 3: 30–50%	8	48,204,958	25	189	Kano	155
Group 4: > 50%	3	14,014,062	7	55	Edo	57
Total	37	195,922,906	100	768		695

HH survey sample				
Malaria endemic group	Selected state	% state population/total population	Sample size planned	Sample size achieved
0–10	Imo	21	80	90
10–30	Gombe	12	45	75
30–50	Kano	51	196	233
> 50	Edo	16	63	81
Total	100	100	384	479

**Fig. 1:**
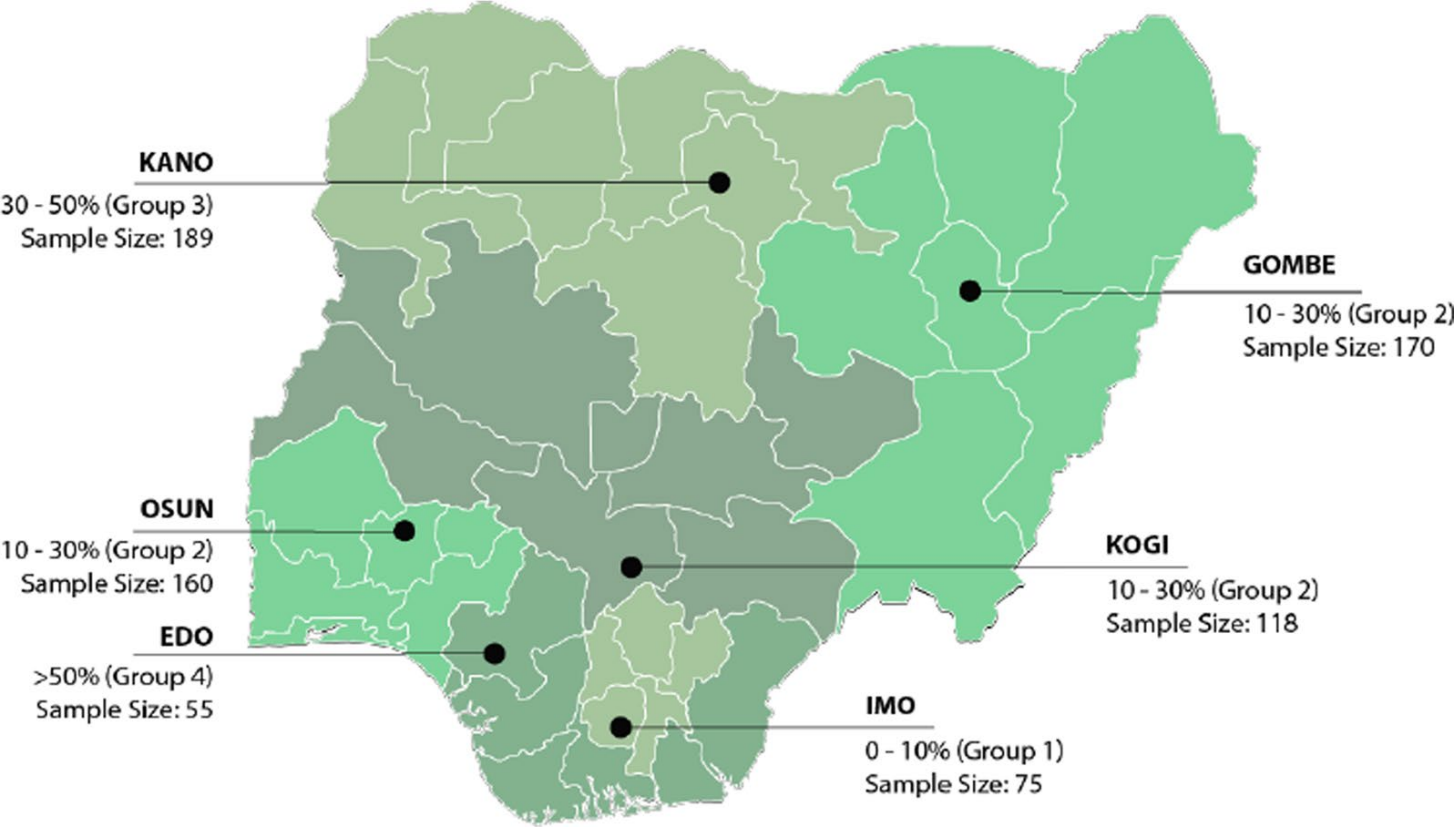
Sampling locations of the outlet survey showing malaria prevalence grouping and outlet sample size

To ensure representation of the six geo-political zones (and thus the different socio-economic and political contexts) in Nigeria, one state from each geo-political zone was selected. Since malaria endemic group 2 represented 50% of the total population of Nigeria three states were allocated for selection to this group and one state for each of the other groups. States were randomly selected and once a state was selected from a particular geo-political zone, that zone was removed from the randomization and the next state selected. The final states selected were Kano, Gombe, Kogi, Imo, Edo and Osun (Fig. 1, Table 1).

Once states had been selected, a list of local government authority (LGAs) was generated for each state and one LGA was randomly selected per state. The second LGA was purposively selected to ensure an urban/rural split and sampling of LGAs that were within a proximate location of one another. For example, if a rural LGA was randomly selected, then a nearby urban LGA was purposively selected, and vice versa. It was expected that urban LGAs would have a greater number of pharmacies relative to PPMVs, while rural LGAs would have greater number of PPMVs. Hence the sample size for each state was split to survey 50% from urban LGAs and 50% from rural LGAs, and within urban sites to include 70% pharmacies and 30% PPMVs, while in rural sites to include 70% PPMVs and 30% pharmacies.

### Demand side analysis: household survey

The objective of the HH survey was to determine HH use of the private sector, demand for ACT medicines, brand preference and factors affecting purchase decision. The survey focused exclusively on low-income households in both urban and rural areas of Nigeria. Purposive sampling of three low-income segments was conducted including HHs with monthly incomes of: (1) NGN ≤ 18,000, (2) NGN 18,001–36,000, (3) NGN 36,001–100,000. For comparative analysis, a small number of samples from HHs with monthly income NGN > 100,000 were also collected.

### Sampling strategy

The total population of the three income segments was unknown, therefore, sample size was calculated assuming an unknown population size, 50% response rate, 5% margin of error and 95% confidence level to derive a sample size of 384 (Table 1).

HHs were selected from four of the six states included in the outlet survey so as to represent each of the four malaria endemic zones in Nigeria and make the survey comparable with the outlet survey. Thus, the state representing Group 2 was randomly selected from the three included in the outlet survey. The final states included for the HH survey were Imo, Edo, Gombe and Kano (Fig. 1). The sample size was distributed across the four selected states according to the same ratio as the size of the state population compared to the total population size of all four states combined (Table 1). The sample per state was planned to be split 50% urban and 50% rural. Purposive sampling was conducted to place 50% of the sample from income segment (1) NGN ≤ 18,000, 35% from income segment (2) NGN 18,001–36,000, and 15% from income segment (3) NGN 36,001–100,000.

### Data collection and quality assurance

PPMV and pharmacy staff completed face-to-face questionnaires with the survey team to collect information regarding anti-malarial stock, price, brand awareness and consumer preference, as well as knowledge of the green leaf logo. Stock information was collected for ACT medicines, both green leaf and non-green leaf, as well as mono-artemisinins and other anti-malarials (for a full brand list asked about in the outlet survey see Additional file 1). Questionnaires were also delivered to heads of households to collect data on diagnosis and treatment-seeking behaviours, ACT brand preference, willingness to pay and knowledge of green-leaf ACT and fake ACT medicines. Both questionnaires were drafted following an extensive literature review and underwent pilot testing before being finalized. Data for both surveys was collected digitally through tablet phones on digitized questionnaires using the LimeSurvey software v2 [19].

Data uploaded to the server were checked daily for consistency and discrepancies. If errors were identified these were immediately notified to the field survey teams to rectify. In addition, quality assurance spot-checks were conducted randomly by survey supervisors to ensure data collection protocols were followed accurately. Random checks were conducted on 10% of all samples. Data was downloaded in excel format and transferred to SPSS [20] and R [21] for analysis.

### Data analysis

Data from the outlet survey were analysed to retrieve proportions and 95% confidence intervals (CI) for key indicators relating to the availability of anti-malarial drugs, brand coverage and consumer preference. Availability was defined as the percentage of outlets with antimalarial/ACT in stock at the time of the survey. Analyses were conducted overall as well as by subgroup, including outlet type (pharmacy or PPMV), region (north or south) or area (urban or rural). Kano, Gombe and Kogi were grouped as northern states and Osun, Edo and Imo were grouped as southern states. Data from the household survey were analysed similarly to gather information on diagnostic and treatment-seeking practices, awareness of ACT brand preference and factors influencing purchase decision, with subgroup analysis by region, area and HH monthly income. Where appropriate, proportions between sub-groups were compared using a binomial test for difference in proportions or a Chi-square test for trend, and p-values significant at the p = 0.05 level are presented.

To assess the impact of the PSCM on different drug types in the market, anti-malarial brands were analysed based on the following grouping: (1) green leaf ACT (i.e., PQ-ACT with the green leaf logo)—these ACT medicines are those directly subsidised under the PSCM, (2) non-green leaf ACT—these ACT medicines contain both PQ and non-PQ ACT, as well as QA and non-QA ACT, but all are ACT medicines that have not been directly subsidised under the PSCM, and (3) non-ACT anti-malarial—which includes both QA and non-QA anti-malarials (see Additional file 2 for a full brand list in each category). It was anticipated that while analysis of green leaf ACT would show the direct impact of the subsidy scheme, the other categories would highlight indirect effects on the wider antimalarial market. For analysis of specific brands, non-green leaf ACT were labelled as those that were PQ or QA versus those that were not since the latter may present an issue for good case management practices (it is thus important to monitor the market for these).

In order to assess the impact of the PSCM over time, anti-malarial and ACT coverage figures among PPMVs and pharmacies were compared to those from previous ACTWatch surveys conducted in 2009, 2011, 2013 and 2015 [16]. Raw data from the ACTWatch surveys were extracted for pharmacies and PPMVs (referred to as ‘drug stores’ in the ACTWatch reports) only in order to make indicators comparable [22]. For each indicator, a Chi-square test for trend was used to indicate any significant change in coverage over time.

### National survey validation workshop

In December 2018, a stakeholder workshop was conducted in Lagos, Nigeria, to share preliminary findings of the 2018 market analysis, including attendance from manufacturers, importers, distributors/wholesalers, pharmacies, PPMVs, as well as officials from the National Malaria Elimination Programme (NMEP), the National Agency for Food and Drug Administration and Control (NAFDAC) and the UK Department for International Development (DFID). Discussions were held to validate findings, provide feedback and identify opportunities and constraints in the market, ensuring utility of key results.

## Results

The final outlet survey sample included 695 outlets including 321 (46.2%) pharmacies and 374 (53.8%) PPMVs. Split by region and area, 59.3% of outlets were from northern states (n = 412) and 65.0% were from urban areas (n = 452). The actual sample size of outlets achieved in the field differed to the planned sample size because of unavailability of pharmacies in rural areas; from a planned 116 pharmacies in rural areas, only 36 were sampled.

Furthermore, only 207 PPMVs were sampled in rural areas compared to the 270 originally planned. Population and coverage of PPMVs and pharmacies was much lower in rural areas than anticipated, thus, the actual sample size achieved is representative of the actual field scenario. In contrast, urban areas achieved a greater sample size than planned due to the higher number of pharmacies and PPMVs available to urban areas than expected, again making the final sample representative of the situation on the ground (285 urban pharmacies were sampled compared to 270 originally planned, and 167 PPMVs were sampled compared to an original 116 planned).

The final HH survey covered a total of 479 HHs nationwide, with 64.3% (n = 308) in northern states and 54.1% (n = 259) in urban areas. The planned sample size was rounded up to 479 in the field for convenience and to account for HH non-response, as well as to include 24 HHs with income NGN > 100,000 per month. The sample size achieved per income segment was 44% NGN < 18,000 (n = 211), 35% NGN 18,001–36,000 (n = 167), 16% NGN 36,001–100,000 (n = 77) and 5% NGN > 100,000 (n = 24).

### Supply-side: outlet survey results

#### Availability of ACT

**Fig. 2:**
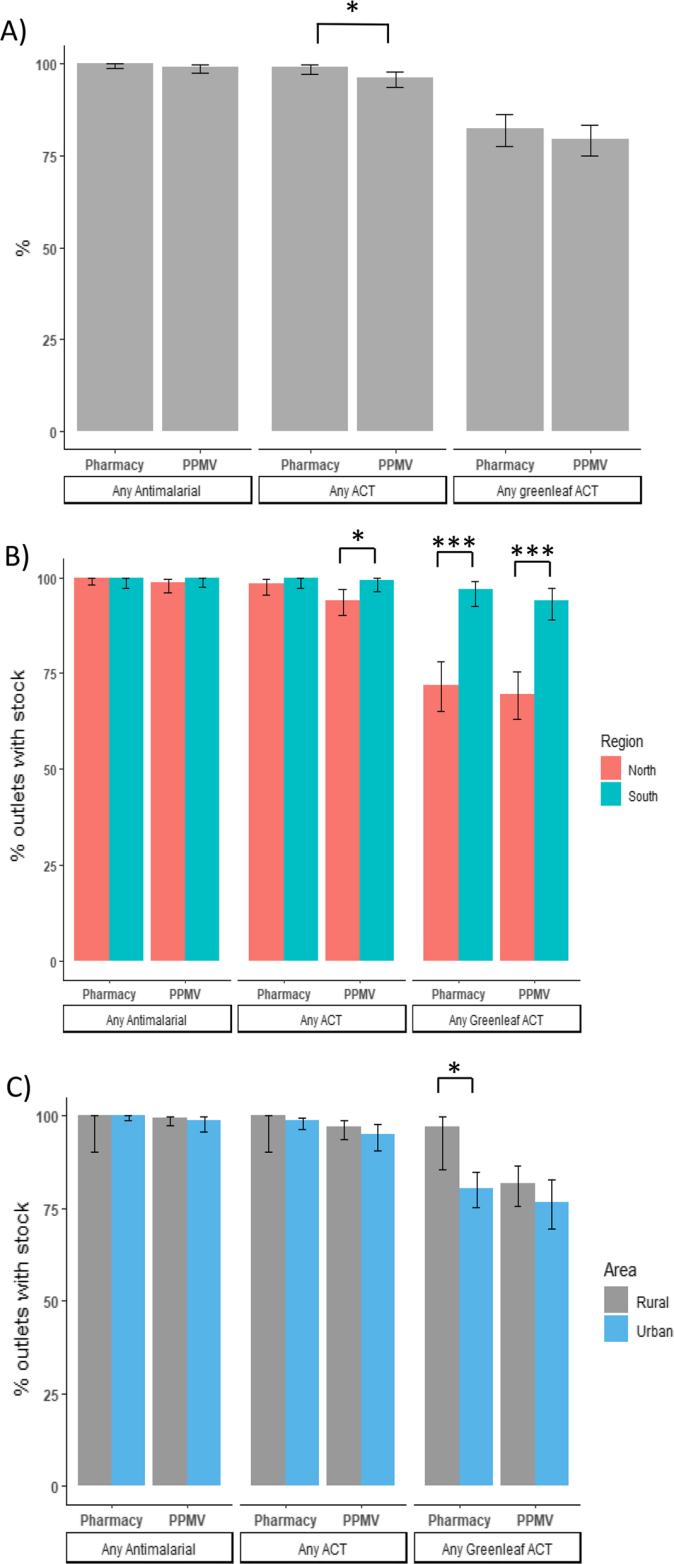
Coverage of different antimalarial drug types in Pharmacies and PPMVs **A** nationwide, **B** by region, and **C** by area. Asterisks show significant differences between sub-groups, *p < 0.05, ***p < 0.001

Outlet availability of anti-malarials (including any anti-malarials) and ACT medicines (including any ACT) was almost universal at 99.6% [95% CI 98.7–99.9] and 97.6% [95% CI 96.1–98.6], respectively, while coverage of green leaf ACT was very high at 80.7%, 95% CI [77.6–83.6] (Fig. 2). There were significantly more pharmacies than PPMVs with ACT medicines in stock (p < 0.05) though there was no difference in the stockage of green leaf ACT (Fig. 2A).

Split by region, availability of ACT medicines was significantly higher in southern PPMVs compared to northern PPMVs (p < 0.05), and coverage of green leaf ACT was significantly higher in the southern states for both outlet types (p < 0.001, Fig. 2B). Green leaf ACT also had significantly higher coverage in rural pharmacies compared to urban pharmacies (p < 0.05, Fig. 2C).

**Fig. 3:**
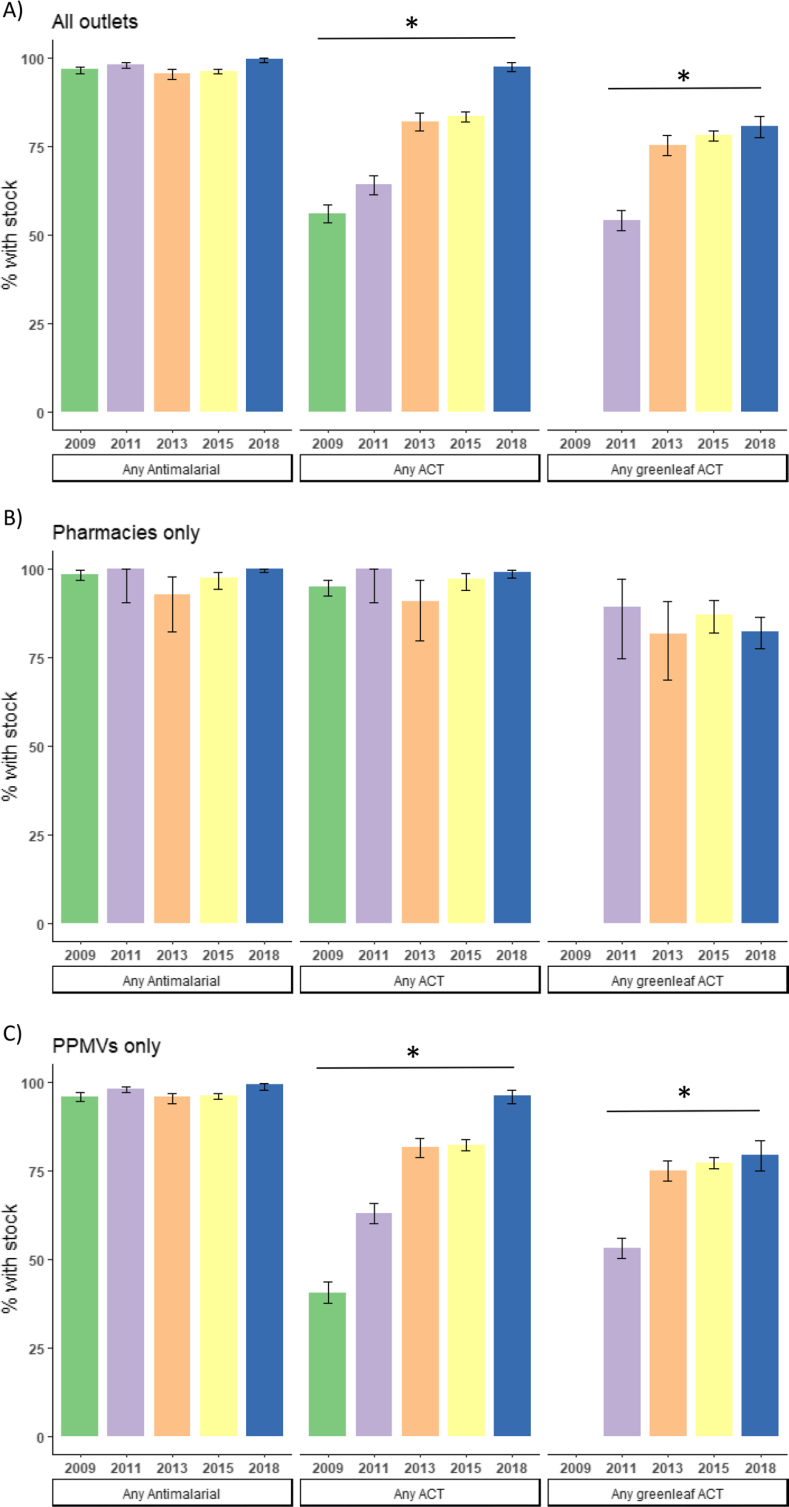
Availability of different antimalarial types across ACTWatch surveys (2009–2015) and the 2018 market survey, including **A** all pharmacies and PPMVs, **B** pharmacies only, and **C** PPMVs only. *test for trend p < 0.001

To determine how availability of the different anti-malarial types had changed over time, availability was compared to previous ACTWatch survey data for pharmacies and PPMVs. Availability of anti-malarials in general had remained high throughout the survey years, but the availability of ACT and green leaf ACT medicines significantly increased from the first ACTWatch surveys in 2009/2011 to the 2018 market survey (Fig. 3). Availability of ACT medicines increased between each survey round from 56.0%, 95% CI [53.4–58.6] in 2009, to 97.6%, 95% CI [96.1–98.6] in 2018 (Chi-square test for trend in proportions: Χ^2^ = 665.86, df = 1, p < 0.001, Fig. 3A). Availability of green leaf ACT medicines increased from 54.2%, 95% CI [51.3–57.0] in 2011 (Greenleaf ACT medicines were not in circulation during the 2009 survey round) to 80.7%, 95% CI [77.6–83.6] in 2018 (Chi-square test for trend: X^2^ = 230.63, df = 1, p < 0.001), although there was a plateauing from 2015 where the availability was 78.0%, 95% CI [76.5–79.5], (Fig. 3A).

Analysis of outlet types separately shows this change over time was among PPMVs, whose coverage of ACT and green leaf ACT medicines increased significantly over the survey years, while pharmacies showed no significant change in any drug category (Fig. 3B, C). Among PPMVs, coverage of ACT increased from 40.5%, 95% CI [37.5–43.6] in 2009, to 96.3%, 95% CI [93.8–97.9] in 2018 (X^2^ = 783.37, df = 1, p < 0.001), and coverage of green leaf increased from 53.1%, 95% CI [50.2–56.0] in 2011 to 79.4%, 95% CI [75.0–83.4] (X^2^ = 213.15, df = 1, p < 0.001) in 2018.

### Availability and market share of specific ACT brands

**Fig. 4:**
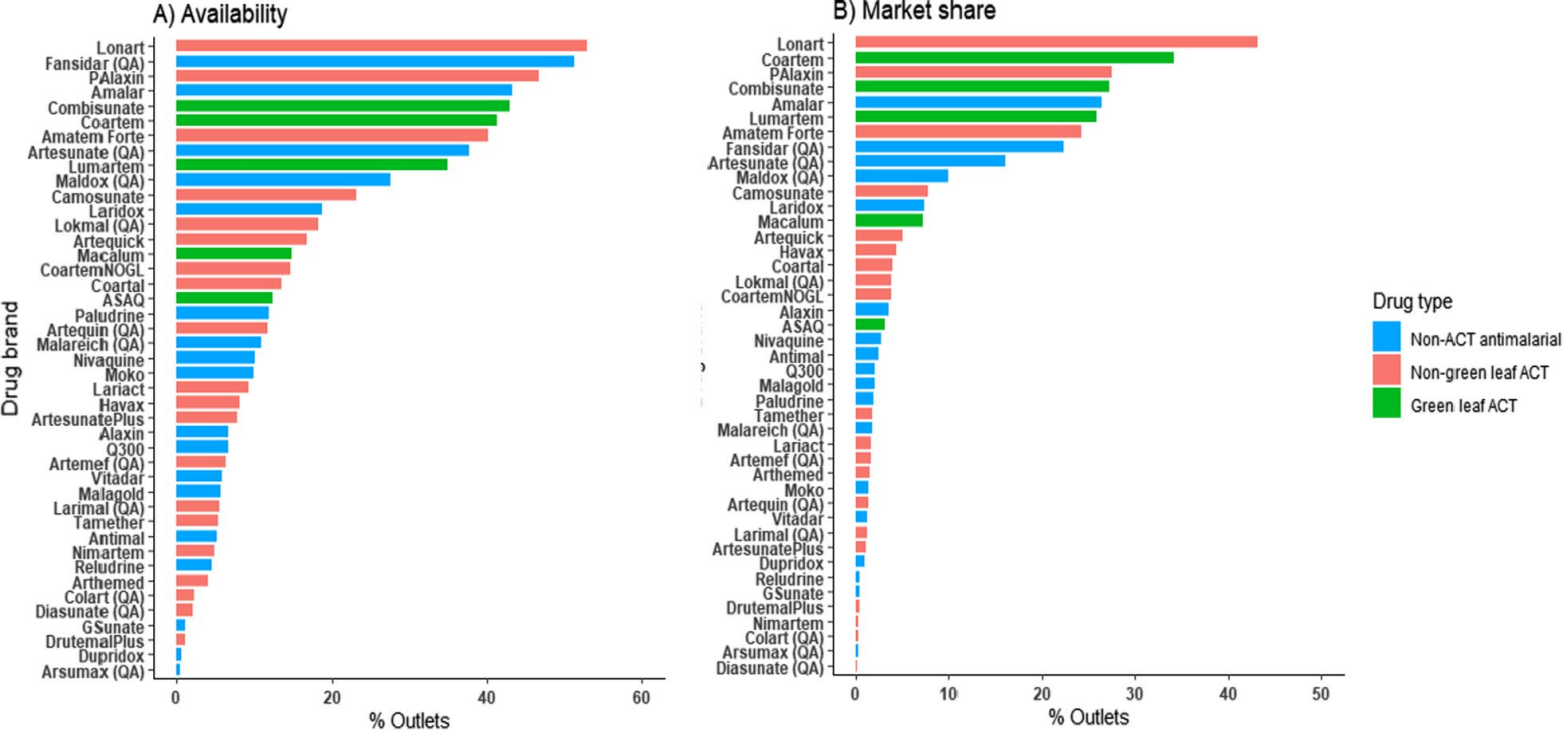
Availability and market share of different antimalarial brands. **A** Availability of brands across outlets on the day of the survey. **B** Market share of brands as reported by outlet staff. Bars show % outlets that named each given brand in the top 5 best-selling brands from their outlet. Includes pharmacies and PPMVs

Among all outlets, the most frequently stocked anti-malarial was the non-green leaf ACT (and non-QA ACT), Lonart (53%). This was followed by the non-ACT anti-malarial, Fansidar (51%) and another non-green leaf ACT (and non-QA ACT), P-Alaxin (47%). The most available green leaf ACT medicines were Combisunate (45%) and Coartem (41%) (Fig. 4A).

Brands top in availability were similar to those reported as top-selling and thus having the highest market share. The non-green leaf ACT, Lonart, was named in the top-5 selling brands of anti-malarial by 43.2% of outlets, followed by Coartem in 34.2% of outlets and P-Alaxin in 27.5% (Fig. 4B). Coartem was followed by Combisunate as the second top-selling green leaf ACT (27.2%). The top-5 selling brands thus included two green leaf ACT medicines, two non-green leaf ACT medicines and one mono-artemisinin (Amalar). Mono-artemisinins made up four of the top-10 brands, however, and four of the top-brands were non-QA.

### Availability and market share of different anti-malarial formulations

Of all the anti-malarials in stock at the time of the survey, the vast majority were in tablet form (88.2%), with some in suspension (4.3%), syrup (5.8%), and dispersible (1.6%) formulations. Of all the tablets in stock, 49.9% were available as double strength dosage (80/480), compared to 34.8% available as standard strength (20/120) and 15.4% as 40/240 strength. Double-strength ACT medicines (80/480) were reported by outlet staff to have greater availability than single-strength ACT medicines (20/120), despite the latter type being the form supplied under the PSCM scheme. Providers estimated that double-strength ACT medicines took up 44% of available stock, compared to 35% for single-strength ACT, 11% for 40/240 dosage drugs and 10% for suspension formulations.

Of the top-5 selling brands, the green-leaf anti-malarials, Combisunate and Coartem, were supplied as standard strength tablets, while Lonart was available as both standard strength and double strength dosages (6 tablets) for adults, as well as in suspension and dispersible forms for children, and P-Alaxin was in a double-strength form (6 tablets) for adults, and suspension and syrup forms for children.

### Sales price

The price of ACT medicines was reported to be volatile by providers, with almost half saying the price would change every quarter or every 6 months (24% quarterly, 24% 6-monthly). Over 60% of providers also stated that the price had been more volatile over the past year compared to the previous year. The Naira devaluation, ACT scarcity and distributor margins were reported as highly significant contributors to the volatility of prices among 75%, 58% and 49% of respondents, respectively.

There was wide disparity in price depending on the brand, type (green leaf, non-green leaf and non-ACT medicines) and pack size (Table 2). Green leaf brands under the PSCM had a median cost of NGN 600, ranging from NGN 350 for Combisunate to NGN 600 for Lumartem, all with 24 tablet formulations. The cost of non-green leaf ACT medicines varied widely depending on the formulation. The standard-strength ACT medicines (24 tablets) had a median cost of NGN 500, making them more affordable than the green leaf ACT, while double-strength dosages (6 or 9 tablets) cost on average NGN 900. The double-strength dosages showed wide variation, however, ranging from NGN 550 for Combisunate DS to NGN 1650 for Coartem DS. The non-ACT anti-malarials were much cheaper with a median price of NGN 180 for three or nine tablets. Overall prices were generally cheaper among PPMVs, in northern states and in rural areas.

**Table 2:** Median price of adult dosages in tablet form among the different antimalarial brands in outlets disaggregated by region (north–south), area (rural–urban) and outlet type (pharmacy-PPMV) as well as by type of antimalarial

Brand name	Type	# tablets in AETD	Median cost (NGN)						
			North	South	Rural	Urban	Pharm-acy	PPMV	Average cost of brand/ type overall
Amalar	Non-ACT antimalarial	3	150	180	200	150	150	180	170
Amalar Plus	Non-ACT antimalarial	9	325	300	400	300	350	300	300
Camosunate	Non-green leaf ACT (ds)	6	600	600	525	600	600	500	600
Coartem	Green leaf ACT	24	350	600	480	600	600	490	500
Coartem DS	Non-green leaf ACT (ds)	6	1650	1600	1700	1650	1600	1800	1650
Combisunate	Green leaf ACT	24	300	400	350	330	330	350	350
Combisunate DS	Non-green leaf ACT (ds)	6	500	550	500	600	600	550	550
Lonart	Non-green leaf ACT (ss)	24	500	500	500	500	500	500	500
Lonart DS	Non-green leaf ACT (ds)	6	1100	1200	1000	1200	1200	1100	1200
Lumartem	Green leaf ACT	24	350	600	600	600	650	600	600
P-Alaxin	Non-green leaf ACT (ds)	9	800	850	800	850	850	800	845
Median by type	All brands		550	650	500	700	718	500	600
	Green leaf ACT		350	600	450	600	600	450	500
	Non-ACT antimalarial		150	200	200	170	155	200	180
	Non-green leaf ACT (ds)		1000	865	800	950	950	800	900
	Non-green leaf ACT (ss)		500	500	500	500	500	500	500

*AETD* adult equivalent treatment dose, *ds* double-strength, *ss* standard-strength

Child dosages were generally slightly cheaper compared to adult dosages and came in a variety of pack sizes (Table 3). In addition, some ACT brands offered child dosages in suspension, dispersible and/or syrup formulations (Table 4). Suspension formulations were more expensive compared to other formulations at an average of NGN 800, but dispersible and syrup formulations were comparable to standard tablets (average cost at NGN 300 and NGN 450, respectively). These alternative forms are not part of the PSCM scheme.

**Table 3:** Median price of child dosages in tablet form among the different antimalarial brands in outlets disaggregated by region (north–south), area (rural–urban) and outlet type (pharmacy-PPMV)

Brand name	Type	# tablets in pack	Median cost (NGN)						
			North	South	Rural	Urban	Pharmacy	PPMV	Average cost of brand overall
Coartem	Green leaf ACT	6	150	150	135	150	150	150	150
		12	200	250	250	225	200	250	250
		18	300	350	300	300	300	300	300
Coartem DS	Non-green leaf ACT (ds)	6	1700			1700	1700		1700
Combisunate	Green leaf ACT	6	110	150	150	140	140	150	150
		12	200	250	250	250	250	250	250
		18	300	300	300	300	300	300	300
Combisunate DS	Non-green leaf ACT (ds)	6	600	600	550	600	600	550	600
Lumartem	Green leaf ACT	6	100	150	110	150	125	135	135
		12	225	250	250	250	250	250	250
		18	300	350	350	350	350	350	350

*ds* double-strength

**Table 4:** Median price of child dosages in alternative forms (suspension, syrup and dispersible) among the different antimalarial brands in outlets disaggregated by region (north–south), area (rural–urban) and outlet type (pharmacy-PPMV)

Brand name	Formulation	Median cost (NGN)						
		North	South	Rural	Urban	Pharmacy	PPMV	Average cost of brand/form overall
Coartem	Suspension	350	350	350	325	350	350	350
	Syrup	400	480	440	450	450	440	440
	Dispersible	400	250	250	300	275	275	275
Lonart	Suspension	800	900	800	850	900	800	850
	Dispersible	225	300	275	300	300	300	300
Lonart DS	Suspension	850	875	850	875	875	850	850
P-Alaxin	Suspension	700	750	700	750	750	700	700
	Syrup		450		450		450	450
Median by form	Suspension	750	800	700	800	800	700	800
	Syrup	400	465	440	450	450	450	450
	Dispersible	250	300	250	300	300	300	300

Compared to previous surveys, the cost of green leaf ACTs increased over the course of the subsidy scheme from 0.91 USD and 0.78 USD in 2011, to 1.47 USD and 1.63 USD in 2018 among pharmacies and PPMVs, respectively (Table 5). Over the same time period, the cost of both standard and double-strength non-green leaf ACT medicines reduced. Standard strength non-green leaf ACT medicines dropped in price by ~ 60% between 2011 and 2018, resulting in a median retail price comparable to green leaf ACT medicines (though, it should be noted this group is only represented by Lonart). Double-strength non-green leaf ACT medicines dropped in price by 10.6% in pharmacies and 17.7% in PPMVs. The retail price of non-ACT anti-malarials was much more volatile and doubled in price between 2011 and 2018, however, this is due to a very low retail price in 2011.

**Table 5:** Median retail price of the different types of antimalarials across the different surveys among pharmacies and PPMVs

	Survey year	Median retail price (USD)^†^		% price change on previous year		% price change 2011–2018 (start–end PSCM)	
		Pharmacy	PPMV	Pharmacy	PPMV	Pharmacy	PPMV
Green leaf ACTs*	2009	na	na				
	2011	0.91	0.78				
	2013	1.59	0.95	**74.7**	**22.3**		
	2015	1.56	1.30	− **1.9**	**36.3**		
	2018	1.47	1.63	− **5.7**	**25.7**	**61.6**	**109.5**
Non-green leaf ACTs (ss only*)	2009	4.55	4.22				
	2011	3.74	3.90	− **17.9**	− **7.7**		
	2013	4.45	3.81	**19.1**	− **2.2**		
	2015	3.38	3.38	− **24.1**	− **11.4**		
	2018	1.63	1.63	− **51.6**	− **51.6**	− **56.3**	− **58.1**
Non-green leaf ACTs (ds only*)	2009	3.77	3.83				
	2011	2.92	3.57	− **22.4**	− **6.8**		
	2013	3.97	3.81	**35.9**	**6.7**		
	2015	3.64	3.64	− **8.4**	− **4.6**		
	2018	2.61	2.94	− **28.2**	− **19.2**	− **10.6**	− **17.7**
Non-ACT antimalarials*	2009	0.52	0.45				
	2011	0.29	0.30	− **43.8**	− **34.3**		
	2013	0.67	0.64	**128.3**	**112.7**		
	2015	0.52	0.52	− **22.1**	− **18.2**		
	2018	0.65	0.59	**25.7**	**13.1**	**123.5**	**96.7**

*ss* standard-strength dosage, *ds* double-strength dosage

*Drug categories include adult dosages of the following:

Green leaf ACT: Coartem, Combisunate, Lumartem

Non green leaf ACT (ss only): Lonart ss

Non-green leaf ACT (ds only): Camosunate, Coartem ds, Combisunate ds, Lonart ds, P-Alaxin

Non-ACT anti-malarials: Amalar, Amalar Plus

^†^Prices in NGN converted to USD using average annual exchange rate in each respective year reported at knoema.com [23]

Among all providers, it was perceived that consumer ACT choice is influenced most by price (33%), followed by the opinion of the pharmacist/PPMV (22%), prior experience using the ACT (21%) and brand (12.5%).

### Demand side: household survey

#### HH treatment-seeking and demand for ACT

**Fig. 5:**
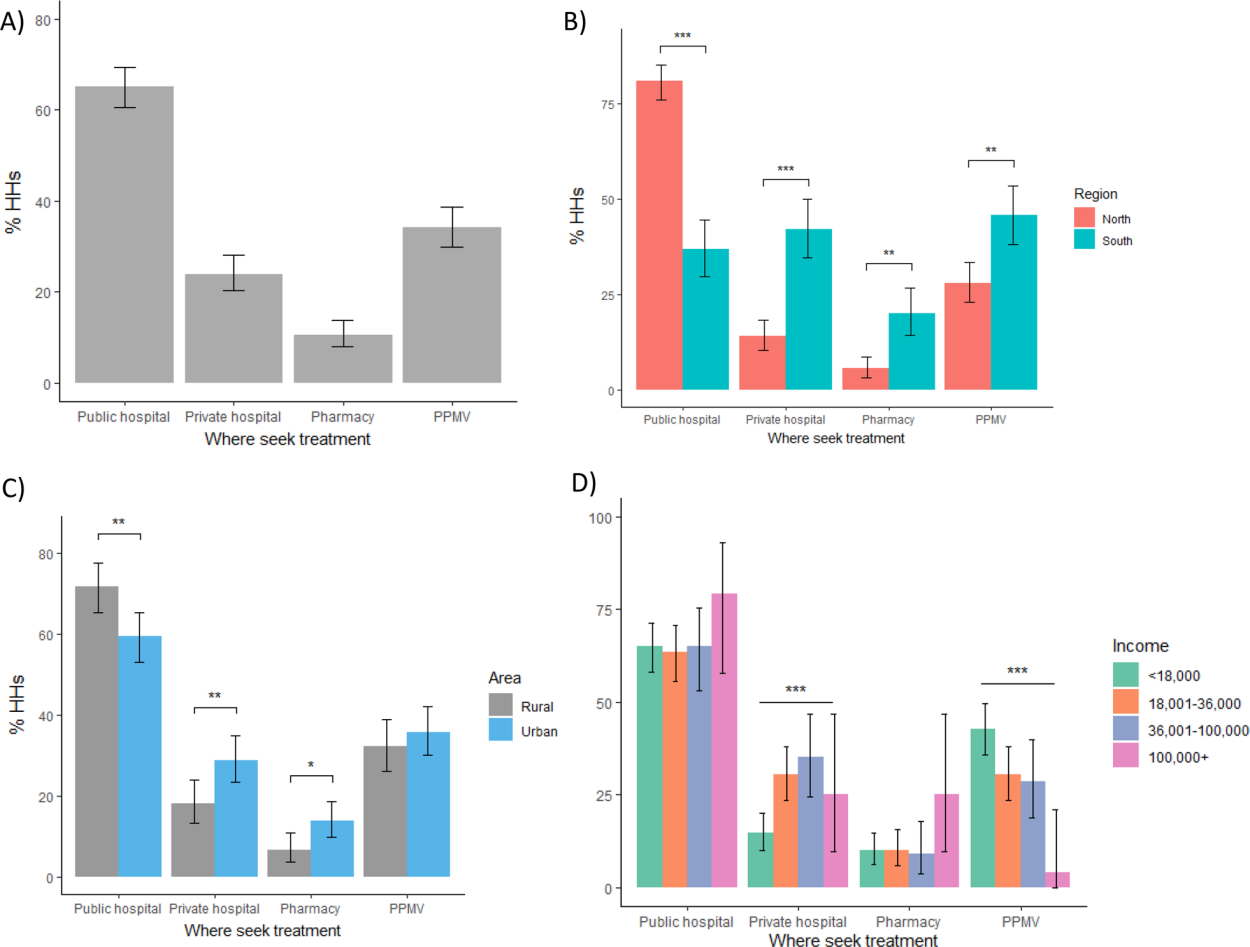
Treatment seeking behaviour following a positive malaria test among HHs **A** nationwide, **B** by region, **C** by area, and **D** by monthly income. Asterisks show significant differences between sub-groups found either by binomial test for difference in proportions or chi-square test for trend: *p < 0.05, **p < 0.01, ***p < 0.001

When asked what they would do if they had a positive malaria test, HHs mostly reported seeking treatment from public hospitals (65.1% [60.6–69.4]), followed by PPMVs (34.2% [30.0–38.7]), private hospitals (24.0% [20.2–28.1]), and lastly, pharmacies (10.6% [8.0–13.8]). Seeking treatment from a public hospital was significantly more common among HHs in northern states (p < 0.001), while seeking help from the private sector including private hospitals (p < 0.001), pharmacies (p < 0.01) and PPMVs (p < 0.01) was greater among HHs in southern states (Fig. 5). Similarly, use of public hospitals was higher among rural households (p < 0.01), while use of private hospitals (p < 0.01) and pharmacies (p < 0.05) was higher among urban households (Fig. 5).

By income level, seeking treatment from PPMVs was higher among lower income households, falling from 43% of HHs in the lowest income segment compared to just 4% HHs in the highest income segment (Chi-square test for trend: X-squared = 16.103, df = 1, p < 0.001). In contrast, no trend by income level was observed for HHs seeking treatment from pharmacies, but 25% of the highest income segment sought treatment from pharmacies compared to just 10% from each of the other income segments, although no pairwise-comparison reached significance due to the lower sample size of the highest income segment.

### ACT awareness and brand choice

Among all households, 62% (n = 299) reported knowing about ACT. Knowledge of ACT was significantly lower in the southern states (42% versus 74%, p < 0.001) and in lower income households (55% in HHs with < 18,000 versus 79% in HHs with 100,000 + income, p-value = 0.03).

HH awareness of green-leaf ACT medicines was 47.0%. These HHs had predominantly heard about green leaf ACT through the hospital (52.9%), followed by PPMVs (23.1%), adverts (22.2%) or pharmacies (20.4%). Among these HHs, the majority (68.4%) stated that they prefer green leaf ACT medicines to non-green leaf ACT medicines, mostly because green leaf ACT medicines are more trusted (76% of HHs that prefer green-leaf ACT), and partly because of lower price (16.9%) or simply that it is the ACT available at the outlet (12.3%).

**Fig. 6:**
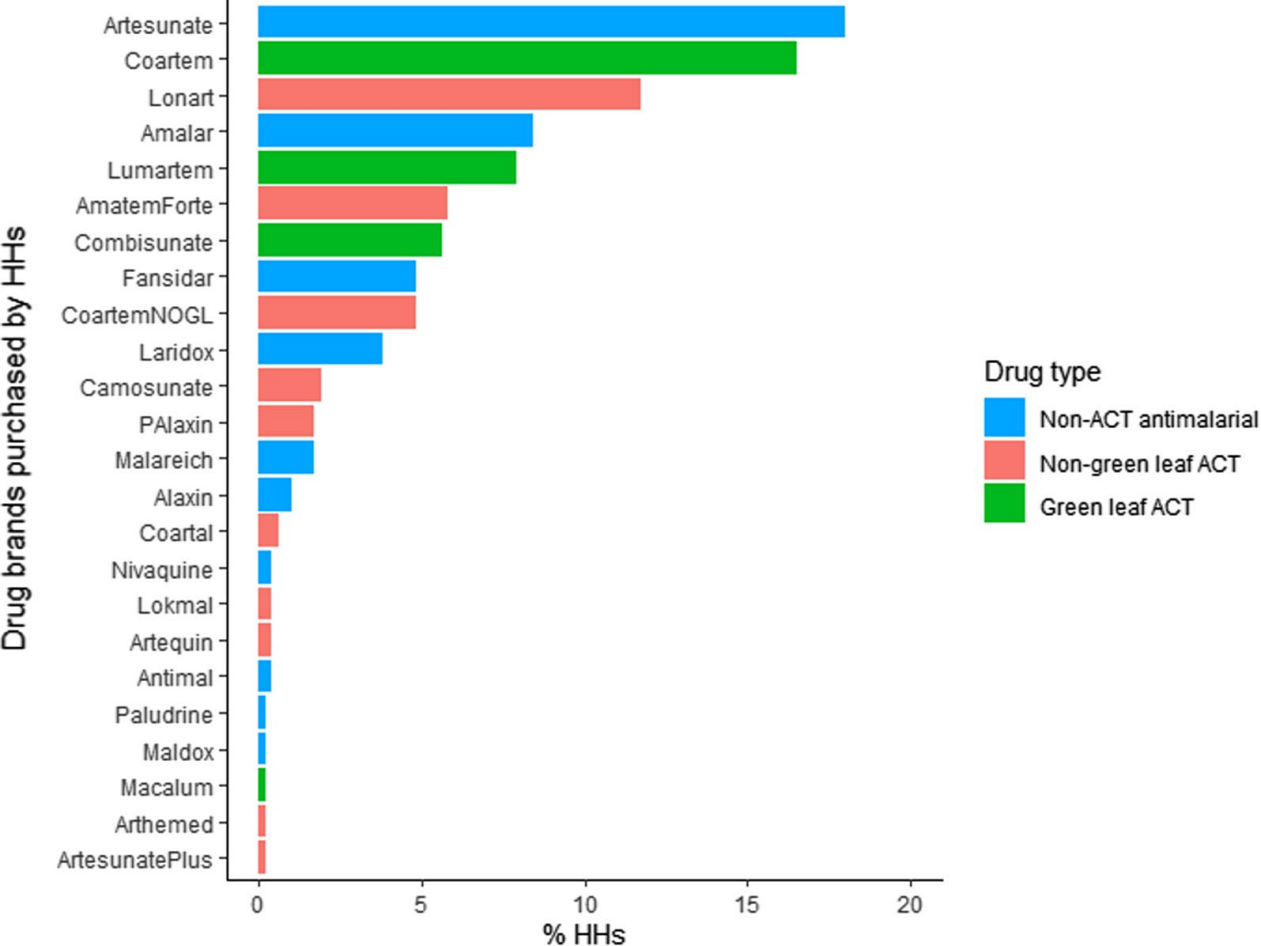
Drug brands purchased by each HH. No HHs reported purchasing: Arsumax, Artemef, Artequick, ASAQ, Colart, Diasunate, DrutemalPlus, Dupridox, GSunate, Havax, Lariact, Larimal, Malagold, Moko, Nimartem, Pamametre, Q300, Reludrine, Tamether, Vitadar

Half of HHs were aware of the brand of anti-malarial that they purchased. The most frequent drug purchased by HHs was the non-ACT anti-malarial, Artesunate (18.0% of HHs), followed by the green leaf ACT, Coartem (16.5%), and then the non-green leaf ACT, Lonart (11.7%, Fig. 6). A similar proportion of HHs reported purchasing green leaf brands (26.3%, 95% CI [22.4–30.5]), non-green leaf ACT brands (23.4%, 95% CI [19.7–27.4]) and non-ACT anti-malarial brands (29.0%, 95% CI [25.0–33.3]). One-third of HHs reported that they frequently change brand (35.5%). The main reason for changing brand was availability (74.1%), followed by price (21.8%) and side effects of the drugs (4.1%).

### Price of ACT medicines and impact on consumer choice

Almost two-thirds of HHs (63.9%) reported that the price of ACT medicines had increased in the previous 12 months, with 50% reporting a greater than 10% increase in price. However, for most HHs this increase did not change their purchase decision and 57.2% will continue to buy the brand they usually buy. Other HHs would request a cheaper ACT medicine (22.2%) or any cheaper anti-malarial (19.0%), and just a few would leave the outlet without buying anything (1.3%).

## Discussion

Analyses of the 2018 market survey, particularly in relation to previous ACTWatch surveys, show a clear impact of the PSCM in terms of increasing ACT availability and affordability across private sector outlets. ACT availability was almost universal among PPMVs and pharmacies, while availability of green leaf ACT medicines was very high (~ 80%). In comparison to previous ACTWatch surveys, the 2018 market survey shows availability of ACT and green leaf ACT medicines significantly increased over the time period of the subsidy scheme. Interestingly, the improvements in availability were among PPMVs as opposed to pharmacies among which availability remained steady, suggesting the scheme had a particular impact in reaching PPMVs. This is important since PPMVs were most frequently visited by lower income HHs and by HHs in rural areas, where the prevalence of malaria is expected to be higher [24–26].

Although supply of ACT medicines had improved, there was significantly lower ACT availability in northern regions (for both outlet types) and in urban pharmacies compared to rural pharmacies. The HH survey also showed differences in public versus private healthcare choice for malaria treatment with HHs in the north favouring the public sector and HH in the south favouring the private sector. It is difficult to speculate why these differences have been observed without more enquiry. Anecdotally, pharmaceutical companies stated that since public distribution programmes had been focused in northern regions, the companies and FLBs had focused their commercial efforts to the south where greater gains could be made (*personal communication, national survey validation workshop).* This could both contribute to improved ACT availability and higher HH demand for these private outlets. Furthermore, northern states have greater levels of poverty compared to southern states [27], and socioeconomic status has been shown to affect choice of healthcare along with other sociodemographic factors including proximity of the health facility, treatment costs, age and level of education [28–32].

The growth in availability of green leaf ACT medicines tailed-off between the 2015 and 2018 surveys, but there was continued growth in availability of ACT overall. This is indicative of market growth of non-green leaf ACT medicines and of the ACT market in general. With the termination of the PSCM in 2018, the decline of growth in green-leaf ACT medicines likely represents a slowing of supply in preparation for the move to an open market. The prominence of non-green leaf ACT medicines can be seen when analysing the availability and market share of specific brands. Non-green leaf ACT medicines had widespread availability and market share comparable to green leaf ACT medicines, and a similar proportion of HHs reported purchasing each type. As well as having a comparable market share, standard-strength non-green leaf ACT medicines were also of a comparable price to green leaf ACT, having had cost reductions over the time period of the PSCM. These cost reductions put them in direct competition with the subsidised green-leaf ACT. Similarly, the cost of double-strength dosages had also reduced. This is a clear indirect effect of the PSCM in driving down the costs of competitors and increasing affordability of ACT medicines in general.

For most HHs, however, price was not the main reason affecting their purchase decision. Most HHs that purchased green leaf ACT medicines did so due to having higher trust in the product, while most HHs in general reported changing brands frequently based on what is available from the outlet at the time. Furthermore, double-strength ACT medicines, despite being much higher in price were reported by outlets as having a larger market share than standard-strength (and cheaper) dosages. This suggests that consumers may be swayed more by convenience than price. As well as different dosages, market share had also been gained by non-subsidised brands via innovation in formulation, particularly for children, with brands offering suspension, syrups and dispersible tablet formulations. Green leaf ACT medicines, on the other hand, were only offered in standard 24 tablet dosages.

A reliance on achieving market share through low-cost means green leaf brands could fail to compete and hold their market share post-PSCM. Since most HHs buy based on availability, there is opportunity for new brands to enter the market, particularly in the absence of competition from green leaf brands. The concern is that this could lead to influx of substandard and/or falsified ACT medicines. New and existing brands need to meet quality standards and be identifiable as having met these standards. However, the current pre-qualification programme is inaccessible to many smaller companies due to high costs and the time needed to complete the registration process [33, 34]. While the increasing coverage of ACT in general is a positive sign, it could also be a cause for concern if local brands increasing their market share are not QA. A high proportion of the top-selling brands were observed to be non-QA but the capacity to rigorously test drug quality in a bioequivalence laboratory does not currently exist in Nigeria.

Of further concern is the continued high availability and market share of oral artemisinin monotherapies (AMT). AMTs were well-stocked and appeared amongst in the top-selling brands, while HHs continued to demand them through their purchase decisions. A contributor to this demand may be that one-third of HHs had not heard of ACT medicines, indicative of a need for effective health communication on the importance of ACT over AMT medicines.

Despite these issues, the PSCM has had a clear positive impact on the ACT market by driving prices down and increasing availability and access. Targeting of the pharmacies and PPMVs through such approaches is important since they make up a significant role in healthcare provision in Nigeria. Private sector outlets, particularly PPMVs, have been reported as the main source for fever treatment-seeking among the general population in repeated national demographic and health surveys [11, 35]. In the 2015 ACTWatch survey, PPMVs were estimated to make up the largest share of the ACT market at 76.0%, while pharmacies took up just 2.5% of the ACT market [16]. In the 2018 market survey, reported use of the private sector was also considerable, with 34% of HHs visiting a PPMV for treatment of a suspect malaria case (higher in southern states and among lower income HHs) and 11% visiting a pharmacist (higher in urban areas and among the wealthiest income segment). As well as supply of artemisinins, these outlets play a significant role in health education since many HHs learned about ACT through these providers. The expansion of public–private-partnerships, such as the PSCM, could thus be key to eliminating malaria in Nigeria and other countries with similar private healthcare provision and demand.

In terms of availability, by 2015, the PSCM showed similar positive results in Kenya, Tanzania and Uganda, but not in Madagascar [12]. Furthermore, although market share of QA-ACT medicines increased in Nigeria, Tanzania and Uganda, there were reductions in Kenya and Madagascar; while prices of QA-ACT medicines reduced in the former three countries and significantly increased in the latter two. The reason for differences between countries is not clear but may be due to effectiveness of implementation, quality of communications and training activities, as well as unstable political contexts [12]. Regular ACTWatch surveys were also conducted in Cambodia where AMFm had been piloted but then discontinued. Despite maintaining high availability of any ACT in both pharmacies and drug stores between 2009 and 2015, the availability of the first-line ACT, ASMQ, significantly declined in both outlet types from 84% in 2009 in both outlets types to just 3% and 12% in 2015 in pharmacies and drug stores, respectively [36].

Despite the 2018 market survey not adhering to the planned sample sizes of outlets in different areas, we are confident the analyses presented here are representative of the situation in the field. When the sampling was planned, the number of outlets in the field was unknown and had to be calculated as such. However, once data collection began it was clear where outlets were fewer or greater in number and sampling was adjusted to reflect the on-the-ground situation. Another limitation of the current study may be in the comparability of the 2018 market survey with the ACTWatch surveys due to differences in methodologies [16, 37]. For example, sampling of LGAs differed due to the objective of each survey; whereas, ACTWatch was designed to capture a comprehensive picture of the anti-malarial market to inform national and international policy, the 2018 market survey was more niche in aiming to assess private sector demand and supply side market factors, hence having a smaller samples of LGAs designed to compare several factors such as urban vs rural, north vs south and PPMV v pharmacy. Since ACTWatch included all public and private outlets, only those data corresponding to PPMVs and pharmacies were extracted for any comparisons. The ACTWatch also included outlets that either had antimalarials in stock on the day of the survey or reported having had them in stock over the previous 3 months, whereas the 2018 market survey did not include this criteria, including outlets even if no stock was or had been present. Finally, ACTWatch did not collect data on specific brands which is a key focus of the 2018 survey and allows insight into how non-green leaf brands responded to increased market competition under the PSCM.

A final limitation is that there is no previous HH survey with which to compare change in HH demand for ACTs and purchasing behaviour. Thus, it cannot be shown whether the PSCM led to an increase in HH demand, knowledge, and ACT choice over time. Finally, focus here has only been on the market for anti-malarials, but a concern with increased supply and demand of ACT medicines may be their overuse and misuse, both of which contribute to poor treatment outcomes due to misdiagnosis and place undue pressure on the malaria parasite to evolve drug resistance [38–40]. The increased demand and supply of ACT should be aligned with diagnostic procedures, including what to do upon a negative malaria test. Case management is a holistic approach including both diagnosis and treatment and thus the focus on one aspect may not be optimal for future similar schemes. Thus, understanding of the malaria RDT and diagnostic market is equally important as the ACT market.

## Conclusions

The subsidy scheme does appear to have had a significant impact on improving availability and affordability of ACT and this is twinned with high consumer demand for malaria treatment from private outlets, particularly among PPMVs. As well as having direct impact through supply of the green leaf brands, the scheme has had indirect effects on the market as a whole, through increasing competition and market share of other, non-green leaf brands. With the end of the subsidy scheme, increased competition for market share can be expected and steps need to be taken to ensure these competing brands are of a high quality, and that the market is kept free of substandard and falsified ACT medicines, and AMT medicines. As well as methods to aid QA-testing of new and existing products, regular monitoring of the post-subsidy, open market is needed such as that seen with bed net distribution programmes [41] and in agriculture [42, 43].

## Supplementary Information

**Additional file 1.** Specific brands asked about in outlet questionnaire.

**Additional file 2.** Categorisation of antimalarial drugs in the analysis of the 2018 market survey.

## Data Availability

The datasets used and/or analysed during the current study are available from the corresponding author on reasonable request.

## Abbreviations

ACT: Artemisinin-based combination therapy
AMFm: Affordable Medicines Facility—Malaria (AMFm)
AMT: Artemisinin monotherapy
CI: Confidence interval
FLB: First line buyer
GFATM: The Global Fund to Fight AIDS, Tuberculosis and Malaria (GFATM)
HH: Household
LGA: Local government authority
PPMV: Proprietary and patent medicine vendors
PQ: Pre-qualified
PSCM: Private sector co-payment mechanism
QA: Quality-assured
RDT: Rapid diagnostic test
