# Supplementary material for: The impact of the private sector co-payment mechanism (PSCM) on the private market for ACT in Nigeria: results of the 2018 cross-sectional outlet and household market surveys
Source: Malar J. 2022 Feb 12;21:42. doi: 10.1186/s12936-021-04039-9 (PMC8841089; doi:10.1186/s12936-021-04039-9)
Supplement: Supplementary file 1 — Additional file 1. Specific brands asked about in outlet questionnaire. [file 12936_2021_4039_MOESM1_ESM.docx]

Specific brands asked about in outlet questionnaire:

| **Coartem** |
| --- |
| **Lumartem** |
| **Asaq** |
| **Combisunate** |
| **Macalum** |
| **Amatem Forte** |
| **Artesunate Plus** |
| **Artemef** |
| **Artequin** |
| **Artequick** |
| **Pamametre** |
| **Coartem (w/outGL)** |
| **Colart** |
| **Coartal** |
| **Larimal** |
| **Havax** |
| **Arthemed** |
| **Lokmal** |
| **Lonart** |
| **Drutemal Plus** |
| **Nimartem** |
| **Camosunate** |
| **Lariact** |
| **P- Alaxin** |
| **Tamether** |
| **Diasunate** |
| **Fansidar/Swidar** |
| **Amalar** |
| **Maldox** |
| **Malareich** |
| **Laridox** |
| **Artesunate** |
| **Arsumax** |
| **GSunate** |
| **Paludrine** |
| **Reludrine** |
| **Antimal** |
| **Malagold** |
| **Moko Liquid Quinine** |
| **Nivaquine** |
| **Q-300** |
| **Dupridox** |
| **Vitadar** |
| **Alaxin** |
| **Others** |
