# Supplementary material for: The impact of the private sector co-payment mechanism (PSCM) on the private market for ACT in Nigeria: results of the 2018 cross-sectional outlet and household market surveys
Source: Malar J. 2022 Feb 12;21:42. doi: 10.1186/s12936-021-04039-9 (PMC8841089; doi:10.1186/s12936-021-04039-9)
Supplement: Supplementary file 2 — Additional file 2. Categorisation of antimalarial drugs in the analysis of the 2018 market survey. [file 12936_2021_4039_MOESM2_ESM.docx]

Supplementary Table. Categorisation of antimalarial drugs in the analysis of the 2018 market survey.

| **Green leaf ACTs** | **Non-green leaf ACTs (without green leaf logo and including both QA and non-QA brands)** | **Non-ACT antimalarials (including both QA ,non-QA brands, PQ and non PQ)** |
| --- | --- | --- |
| Combisunate | Coartal | Alaxin |
| Coartem | Diasunate (QA, Emzor) | Amalar |
| Lumartem | Drutemal Plus | Artesunate (QA) |
| ASAQ | Lokmal (QA, Emzor) | Antimal |
| Macalum | P-Alaxin | Dupridox |
|  | Nimartem | Fansidar (QA, Roche) |
|  | Havax | Paludrine |
|  | Amatem Forte | Laridox |
|  | Tamether | Malareich (QA, Zentiva Div of Sanofi) |
|  | Pamametre | Vitadar |
|  | Lariact | Moko |
|  | Artequin (QA, imported by Occulus) | Reludrine |
|  | Artequick | Malagold |
|  | Camosunate | Arsumax (QA, Sanofi) |
|  | Artesunate Plus | Gsunate |
|  | Arthemed | Maldox (QA, Emzor) |
|  | Colart (QA,GSK) | Q300(Quinine Sulphate Tablets) |
|  | Coartem nogl | Nivaquine |
|  | Lonart |  |
|  | Larimal (QA) |  |
|  | Artemef (QA) |  |
|  |  |  |
